# Post-acute infection: before we forget it again

**DOI:** 10.1093/cei/uxag061

**Published:** 2026-10-26

**Authors:** Helena Borland Madsen, Claus Desler

**Affiliations:** Department of Biomedical Sciences, University of Copenhagen, Copenhagen, Denmark; Department of Biomedical Sciences, University of Copenhagen, Copenhagen, Denmark

**Keywords:** post-acute sequelae, Long COVID, inflammation, mitochondrial dysfunction, infectious diseases

## Abstract

Long COVID did not invent the post-acute phase of infection; it made it impossible to ignore. Acute infections are still often framed as time-limited events that end in recovery, deterioration or death, but this view fails to capture patients who remain biologically and clinically unwell after the acute infection has passed. The problem extends beyond SARS-CoV-2. Post-acute morbidity has been described after influenza, Epstein–Barr virus infection, Q fever, sepsis, and other infections, yet definitions, coding systems, and clinical pathways remain fragmented. We argue that post-acute infection should not be organized only by the initiating pathogen. Such pathogen-centric labels may be clinically tidy, but they risk producing heterogeneous cohorts, diluted biomarker signals, and disease-specific silos that obscure shared mechanisms of incomplete recovery. Instead, the field needs a second axis: the host-recovery state. Persistent inflammation, immune remodelling, loss of tolerance, antigenic stimulation, endothelial dysfunction, mitochondrial impairment, and tissue injury may represent overlapping immunometabolic failure modes across infections. Defining these states over time could help transform post-acute infection from a residual clinical label into a mechanism-informed framework for research, biomarkers, and care.

## Teaser

Long COVID did not invent the post-acute phase of infection; it made it impossible to ignore. Acute infections are still often framed as time-limited events that end in recovery, deterioration or death, but this view fails to capture patients who remain biologically and clinically unwell after the acute infection has passed. The problem extends beyond SARS-CoV-2. Post-acute morbidity has been described after influenza, Epstein-Barr virus infection, Q fever, sepsis and other infections, yet definitions, coding systems and clinical pathways remain fragmented. We argue that post-acute infection should not be organised only by the initiating pathogen.

## Graphical Abstract

**Graphical Abstract:**
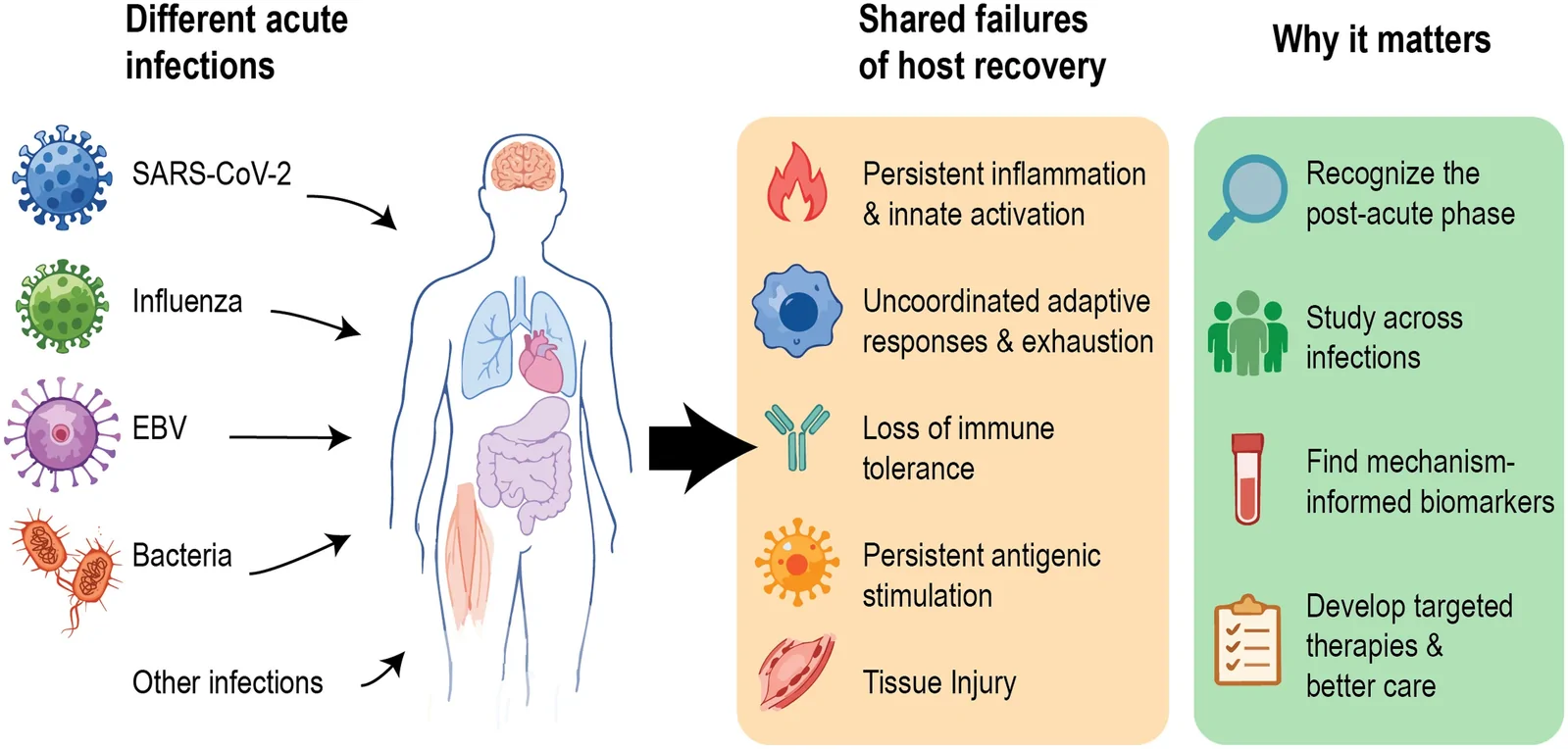

## Introduction

Acute infections are often treated as time-limited events that end in recovery, deterioration, or death. This view is clinically convenient, but biologically incomplete and the post-acute phase is often overlooked. The SARS-CoV-2 pandemic exposed a major blind spot in the knowledge about post-acute sequelae of infectious diseases and identified the need for more knowledge on the period to be translated into better clinical care for patients [1]. Depending on population, variant, vaccination status, and case definition, persistent symptoms compatible with Long COVID have been reported in approximately 10–20% of individuals infected with SARS-CoV-2 [2, 3]. These symptoms are associated with reduced health-related quality of life [2], increased risk of later cardiovascular, kidney, and pulmonary disease [4], and increased healthcare costs after the acute phase [3]. Yet the core lesson from Long COVID should not be that SARS-CoV-2 is uniquely capable of generating prolonged disease. Rather, it should force us to ask whether post-acute morbidity has been systematically underestimated across infectious diseases. Severe post-infectious conditions are increasingly recognized after multiple infections [5]. And not only rare or exotic pathogens. Influenza, one of the most common acute infections encountered by health systems, may also carry an under-recognized post-acute burden; comparative cohort studies suggest that, after non-severe illness, SARS-CoV-2 confers only a modestly higher post-acute risk than influenza [6]. The challenge for clinicians, researchers, and health systems is therefore 2-fold: first, to recognize the post-acute phase as part of the disease trajectory itself, not merely the aftermath of infection; and second, to define and study it in ways that avoid splintering post-infectious care and research into pathogen-specific silos, because siloed labels may be clinically tidy, but they risk obscuring shared mechanisms of incomplete recovery.

## Post-acute phases, same same, but different

The post-acute phase is not a modern concept. Several historical and modern sources describe prolonged symptoms going back more than a century [7]. An example is the 1918 influenza pandemic, commonly known as the Spanish flu, where the post-acute phase was described as including fatigue, weakness, neuropsychiatric symptoms, sleep disturbance, dizziness, and reduced ability to work. A 2024 public health review notes that it is difficult to quantify Spanish flu sequelae, but cites contemporary English estimates suggesting that, among 1000 influenza cases, around 800 recovered without sequelae, around 70 died, and around 130 did not fully recover or develop sequelae [7]. That should be treated cautiously, but it is strikingly close to the broad idea that a substantial minority experienced post-acute morbidity.

Modern infectious disease research also provides several examples of post-acute morbidity beyond SARS-CoV-2 and influenza. Post-infective fatigue syndromes are well described after Epstein–Barr virus (EBV) infection [8], and prolonged fatigue after acute Q fever is reported in roughly one-fifth of symptomatic *Coxiella burnetii* infections [9]. Other viral infections, including herpesvirus infections, have long been linked to post-infectious neurological and systemic syndromes, although the strength of evidence varies by pathogen and phenotype [10]. A pre-COVID-19 estimate indicated that the incidence of the 31 most common infections was 7577 cases per 100 000 population in the EU [11], corresponding to 30–35 million episodes per year in the EU with influenza having the highest burden (30%) [11, 12]. This scale matters. If even a small proportion of common infections lead to prolonged symptoms, organ complications, or immune-mediated disease, the post-acute phase may represent a substantial but under-measured contributor to chronic disease burden.

## From clinical label to biological black box

Long COVID was not the first post-acute infection syndrome to be named, but it was the first to force post-acute infection morbidity into mainstream clinical, research and public-health attention at global scale. Long COVID has been essential for naming a previously neglected clinical reality. Yet as a mechanistic category, it is increasingly unhelpful. The term now covers a broad landscape of persistent symptoms, new diagnoses, organ complications, and relapsing multisystem disease after SARS-CoV-2 infection. This breadth is clinically necessary: without inclusive definitions, many patients would remain invisible to health systems. But what is inclusive at the clinic door can become obstructive at the laboratory bench.

The terminology itself reflects the problem. Post-COVID condition, post-COVID-19 syndrome, COVID-19 sequelae, post-acute sequelae of SARS-CoV-2 infection and Long COVID are often used interchangeably, although they are not always operationalized in the same way. The World Health Organization defines post-COVID-19 condition as symptoms occurring usually three months after probable or confirmed SARS-CoV-2 infection, lasting at least 2 months and not explained by an alternative diagnosis [13]. The National Institute for Health and Care Excellence in the UK separates ongoing symptomatic COVID-19 from 4 to 12 weeks and post-COVID-19 syndrome beyond 12 weeks [14], whereas the National Academies of Sciences, Engineering, and Medicine in the USA defines Long COVID as an infection-associated chronic condition present for at least 3 months as a continuous, relapsing-remitting, or progressive disease state affecting one or more organ systems [15]. These definitions are all useful, but they do not solve the same problem. They variously prioritize clinical recognition, care pathways, public-health surveillance, patient inclusion, and research standardization. The difficulty is that, in practice, the resulting terms are often treated as interchangeable labels for the same entity.

Consequently, differences in how these definitions are operationalized are not merely semantic. Different definitions thus identify different patient groups, leading to substantially different prevalence estimates. A 2025 JAMA Network Open cohort study showed that applying these definitions produced substantially different prevalence estimates, with Long COVID among COVID-positive participants ranging from 32 to 42% at 3 months and 15 to 22% at 6 months [16]. Broad case definitions create a double risk: they may group biologically distinct processes under the same label, while narrower definitions may miss patients with genuine post-infectious pathology if symptoms fall outside predefined criteria, for example if acute infection was undocumented, or if the presentation falls outside a chosen time window.

The International Classification of Diseases (ICD) system codifies the same trade-off. U09.9 (‘Post COVID-19 condition, unspecified’) is useful as a linkage code connecting later morbidity to prior COVID-19, but it does not define underlying pathology. This is not a minor technical issue. If the post-acute phase is defined primarily by broad symptom clusters and non-specific linkage codes, cohorts will inevitably combine patients at different biological stages of recovery and with different mechanisms: early inflammatory persistence, delayed organ injury, immune exhaustion, autonomic dysfunction, metabolic dysregulation, cognitive dysfunction and, unavoidably, unrelated comorbidity, may all be counted under the same post-COVID label. Conversely, relevant cases may be excluded because they fall outside a symptom template or a timing definition. In practice, definitional noise flattens a dynamic disease trajectory into a single residual category after infection.

The coding landscape for other post-acute infections underscores that COVID-19 is, if anything, the best-case scenario. Comparable post-acute conditions after influenza, EBV, bacterial pneumonia, or sepsis are generally not captured by pathogen-specific post-acute codes. Instead, they are scattered across broad ICD categories such as G93.31 (‘postviral fatigue syndrome’), G93.39 (‘other post-infection and related fatigue syndromes’), or B94.8/B94.9 (‘sequelae of other specified’ or ‘unspecified infectious and parasitic diseases’). The recent 2019 updated ICD-11 carries the same taxonomy forward almost unchanged: 8E49 (‘postviral fatigue syndrome’) is still not a pathogen-specific post-acute framework, and B94.8/B94.9 simply become 1G8Y/1G8Z, the same ‘other/unspecified’ catch-all. In principle, postcoordination in the ICD-11 update can link a sequela code to its antecedent infection, but this depends on documentation discipline and on clinicians recognizing the presenting symptoms as infection-related, which is still problematic to date [17]. Outside COVID-19, we are therefore even worse prepared to study the post-acute phase as a disease trajectory. A patient with persistent fatigue after influenza, organ dysfunction after sepsis, neurocognitive symptoms after EBV, or delayed cardiopulmonary disease after bacterial pneumonia may be coded by symptoms, by organ involvement, by generic infectious sequelae, or not coded as post-infectious at all. If post-acute syndromes are defined only by pathogen history and persistent symptoms, we will reproduce the same problem across diseases: heterogeneous patients, noisy cohorts, diluted biomarker signals and mechanistic studies that cannot distinguish failed resolution, immune exhaustion, tissue damage, or metabolic dysfunction. In this landscape, Long COVID is not the endpoint of the problem; it is the best-coded example of a much larger failure to define recovery after infection.

## Gotta catch ‘em all

The initiating infectious diseases differ in tissue tropism, acute immune activation, pathogen persistence, disease severity and affected populations. It is therefore natural that their post-acute phases are first understood through the lens of the original pathogen. However, this pathogen-centric logic risks reproducing the very blind spot that Long COVID exposed: post-acute morbidity becomes divided into infection-specific silos, each with its own definitions, cohorts, outcomes and care pathways. In practice, this can mean that the same biological problem is followed in entirely different clinical and research lanes depending on whether it began as influenza, SARS-CoV-2, EBV, bacterial pneumonia, or sepsis, fragmenting both follow-up and mechanistic discovery. This fragmentation makes it difficult to identify shared risk trajectories, compare outcomes across conditions or translate mechanistic insight into broadly applicable clinical tools [1, 18]. Yet the host may not have unlimited ways to fail recovery. Recent post-infection research points to recurring biological themes across post-acute outcomes, including chronic inflammation, dysregulated immune responses, endothelial dysfunction, mitochondrial impairment, and maladaptive repair [5], illustrated in Fig. 1. These shared pathways do not erase pathogen-specific biology, but they suggest that different infections can converge on overlapping host-recovery states. The opportunity is therefore to add a second axis to post-acute infection research: not only which pathogen initiated the disease, but which recovery programme failed. Such a framework could help identify high-risk individuals across infections, define cross-condition biomarkers, and support mechanism-informed interventions that are not trapped within a single pathogen label.

**Figure 1:**
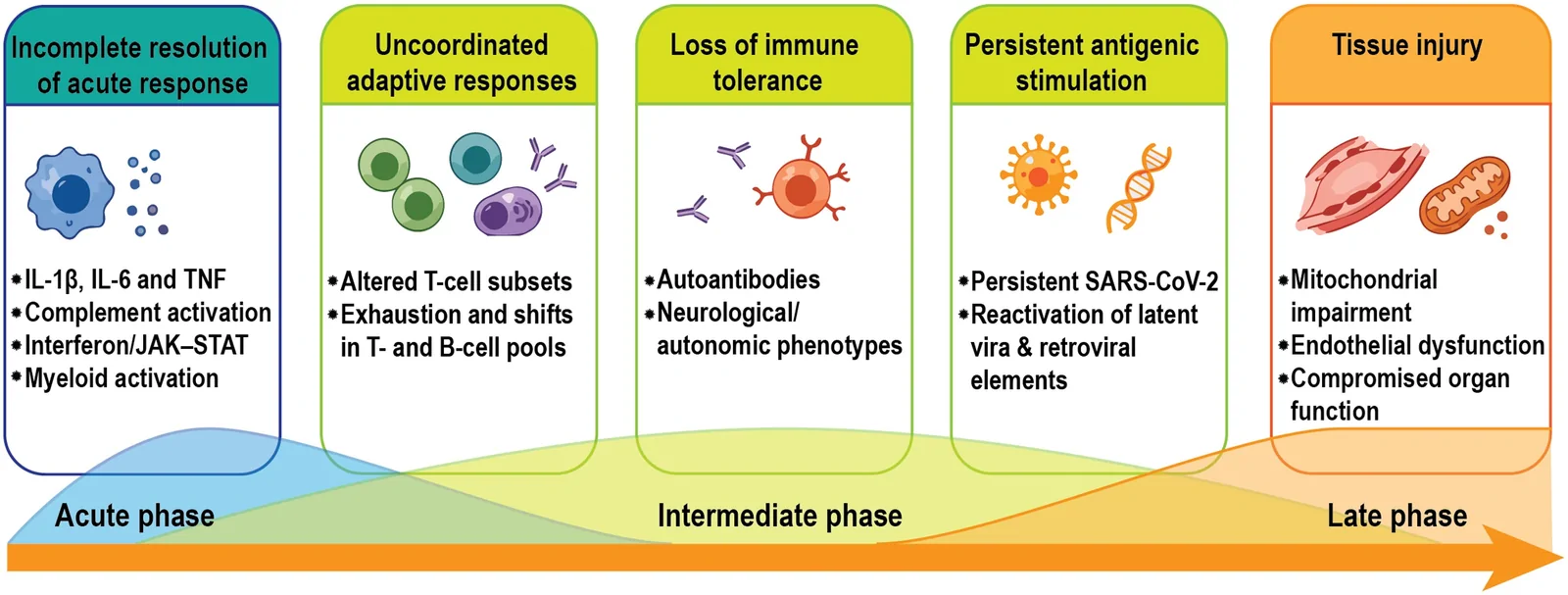
The post-acute phase over time after infection. In the acute phase, the initial response to infection might not be properly resolved with persistent innate immune inflammatory signalling. The intermediate phase can be quite diverse with a subset presenting with uncoordinated adaptive immune responses, autoimmunity, and/or persistent antigen. The late phase is shaped by the previous phases and often dominated by mitochondrial and endothelial dysfunction and tissue damage. The proposed sequence is expected to apply most closely to non-severe post-infectious trajectories. In severe acute illnesses such as sepsis, tissue injury occurs during the acute phase and the post-acute phase may instead reflect incomplete repair and adaptation.

Across post-acute infection syndromes, the immune signal may not be static, and this may be part of why mechanistic studies so often disagree. Early after infection, the most visible signal may be incomplete resolution of the acute host response: persistent innate inflammatory signalling, reflected by cytokines and chemokines (including IL-1β, IL-6, and TNF networks in some Long COVID cohorts [19, 20]), complement activation, interferon/JAK–STAT programmes and myeloid activation. In other patients, however, the more informative signal may not be soluble inflammation at all, but a reshaped immune system resulting in uncoordinated adaptive responses: altered T-cell subset distributions, exhausted antigen-specific CD8 T cells, shifts in memory B- and T-cell pools and myeloid skewing [21]. For a subset, the biology may move further into loss of immune tolerance. Autoantibodies have been reported in Long COVID [22], including GPCR- and RAS-related targets and antibodies linked to neurological or autonomic phenotypes, although their diagnostic value and causal role remain unresolved and may represent an endotype rather than a universal mechanism. Another subset may be driven by persistent antigenic stimulation: ongoing SARS-CoV-2 antigen, reactivation of latent herpesviruses such as EBV and possibly activation of endogenous retroviral elements such as HERV-W [23, 24]. The details here are still being worked out, but the implication is important: an apparently ‘post-infectious’ state may continue to receive immune stimulation long after the acute illness is considered over. Finally, these immune outcomes intersect with tissue repair and immunometabolism. Complement activation, endothelial dysfunction and mitochondrial impairment may convert immune dysregulation into persistent symptoms or organ-specific disease [25, 26]. Much of the current literature on mitochondrial dysfunction derives from bulk peripheral blood mononuclear cell analyses, where changes in immune-cell composition may contribute to observed bioenergetic differences without implying cell-intrinsic mitochondrial defects, adding another layer of heterogeneity to the same problem. Furthermore, a lot of the available evidence for the temporal ordering proposed here, derives from cross-sectional studies using different cohorts and case definitions, and apparent temporal differences may therefore reflect differences in sampling rather than biological progression. Longitudinal cohort studies are warranted in the future to test whether these proposed host-recovery states represent true sequential transitions.

Although the precise temporal ordering remains to be established, the available evidence suggests that the post-acute phase is better viewed as a series of overlapping immune states than as a single, static condition. It may be a sequence of overlapping states: early incomplete resolution dominated by soluble inflammatory mediators; an intermediate phase shaped by persistent antigen, autoimmunity or latent virus reactivation; and a later phase in which immune remodelling, exhaustion, trained-immunity-like reprogramming and tissue damage become more informative.

The post-acute phase is therefore not merely ‘what remains’ after infection. It is a biological transition zone. Some patients complete the transition and restore immune-metabolic homeostasis; others remain trapped in states of inflammatory persistence, immune exhaustion, viral or antigenic stimulation, autoimmunity, endothelial dysfunction, mitochondrial impairment or maladaptive repair. These states may overlap, evolve, and differ between patients, but they provide a more useful framework than either symptom lists or pathogen labels alone. This also changes the biomarker question. The field should stop searching for a single Long COVID biomarker, or a single post-influenza biomarker, as if each pathogen produces one uniform post-acute disease. Instead, we should ask which markers define failed resolution, persistent innate activation, immune remodelling, loss of tolerance, viral reactivation, tissue damage or mitochondrial dysfunction, and when these markers are most informative during recovery. While the initiating pathogen remains important for identifying pathogen-specific drivers that may be therapeutically actionable and diverse across pathogen species, the proposed host-recovery axis instead provides a framework for stratifying patients according to the dominant biological process. Together, pathogen and host state may guide both causal therapies and mechanism-directed interventions synergistically.

In this view, Long COVID is not the endpoint of post-acute infection research. It is the proof of concept. It shows that post-acute morbidity can be common, disabling, costly, and biologically complex. But it also shows the danger of broad labels without mechanistic subdivision. If the field now builds separate post-acute silos for every infection, we will repeat the same mistake at larger scale. The alternative is to define post-acute infection as a dynamic immunometabolic disease space: initiated by different pathogens, shaped by different host factors, but converging on recurring failures of immune, metabolic, and tissue recovery. That is where the next generation of cohorts, biomarkers, and interventions should be built.

## Data Availability

Not applicable.

## Abbreviations

EBV: Epstein–Barr virus
ICD: The International Classification of Diseases
